# Progress of Cholera

**Published:** 1849-06

**Authors:** 


### PROGRESS OF CHOLERA.

The latest medical journals from France are filled with details respecting the progress of cholera in that country. Thus far, the epidemic has assumed a much milder form than that in which it appeared in 1832. The invasion appears to have taken place as far back as the 20th of last October. On the first of March the four departments of the north presented 1,692 cases and 803 deaths. In one of these departments alone, in 1832, there occurred, from the 14th of April to the 1st of December, 12,557 cases, and 6,040 deaths; while for three months, this year, the number of cases has been only 378, and the deaths but 222. The symptoms are of much less violence than they were in 1832, and the typhoid character predominates.

The two first cases of cholera occurred in Paris on the 9th of March, and out of the first eighteen, twelve proved fatal. But it is remarked that these victims to the disease "were either laboring under chronic affections of long standing, or confirmed drunkards, or living in the most wretched state of filth and want." In Paris, the difference between the epidemic of 1849 and that of 1832 is as striking as in the provinces. In the space of seven days, in the latter year, there were recorded, in that city, several thousand deaths; whereas now the number of cases in the same lapse of time is but eighteen, and the deaths twelve. On the seventeenth day of its reign it had carried off 161 persons; on the seventeenth day after it broke out in 1832 it had been fatal in 7,000 cases. Up to the 30th of March, 623 cases and 317 deaths had occurred in the hospitals; and the sum total for the whole country, including the capital, from the 25th of September to the 27th of March, has been 3,062 cases, and 1,445 deaths. In 1832, in the same period, forty-one departments were invaded, instead of seven, as now, and in four months there were no fewer than 120,000 cases and 60,000 deaths. How far this improvement depends upon the season of the year, or upon the fact, as suggested by a French journal, "that the epidemic is, in some degree, *worn out,*" remains to be seen.

The greatest mortality in the hospitals has been in Salpetriere, an asylum for indigent females of advanced age, and for the insane of the same sex, of which it contains upwards of 5,000. Up to the 30th of March, 209 cases and 126 deaths had occurred there. In all the hospitals of Paris, up to that time, 623 cases had been presented, of which 317, or more than one-half, were fatal.

The cases and deaths in Great Britain up to the 21st of March are stated to have been as follows: London, 1,205 cases, 617 deaths. Provinces, 1,077 cases, 476 deaths. Scotland, 12,640 cases, 5,219

deaths. During the last week of March there were only ten fatal cases of cholera in the metropolis and its environs, and official reports of cases and deaths are no longer made, public apprehension having subsided as to the immediate danger; but there are many who are not without fears that, as in 1832, another outbreak may occur in the summer.

The epidemic broke out suddenly at Limerick, in Ireland, in March last, and more than a thousand, out of a population of 60,000, were swept away by it before the 7th of April. All the cases for the first few days, it is stated, "came from one locality, the resort of drunken sailors, abandoned women, and people of that class; and some of the most marked and fatal cases happened in crammed up lodging-houses during the Assizes, which were holding at the time."

In New Orleans, the list of interments from cholera, from the 17th of February to the 14th of April, number 1,008. The citizens had begun to congratulate themselves that the epidemic had passed away when it broke out a second time with increased violence. The following table, given by Dr. Crossman, in the New Orleans Medical and Surgical Journal for May, shows the gradual rise and declension of the disease for several weeks:

| | Cholera. | Total. |
|---|---|---|
| Deaths for the week ending 3d March, | 25 | 112 |
| " " " " 10th March, | 50 | 162 |
| " " " " 17th March, | 116 | 296 |
| " " " " 24th March, | 287 | 421 |
| " " " " 31st March, | 238 | 378 |
| " " " " 7th April, | 117 | 228 |
| " " " " 14th April, | 73 | 197 |
| " " " " 21st April, | 101 | 232 |

The mortality was greatly swelled by the immigrants who were pouring into the city by almost every arrival from sea. Comparatively few resident citizens perished. The pestilence was fast declining at the last dates, but as all the causes favorable to its renewal are now in active operation it can hardly be hoped that it has finally disappeared from that city.

In St. Louis during a good part of May the number of deaths from cholera was said to be about twenty. Four cemeteries reported twenty interments from the disease on the 25th, and some others failed to report. The immense number of immigrants arriving there contributed largely to the mortality. In many of the towns on the Mississppi above, the disease has prevaled to a considerable extent, and has proved fatal to a number of persons on their way to California.

The epidemic has increased at Cincinnati, and a number of deaths are daily announced.

At Nashville for thirty days before the 20th of May, the date of our last letter from that city, no case of the disease had occurred; but two days later it reappeared, and six fatal cases occurred in a short time.

At Maysville there have been several fatal cases; and four deaths from cholera, in the Lunatic Asylum at Lexington, were reported about the 22d of May. No case had appeared among the citizens. On the last night in April several deaths from the disease occurred in Louisville, and early in May a few fatal cases were reported in Jeffersonville and New Albany. None have occurred for several weeks past in either of the latter places.

In this city, occasional cases are still reported, but the epidemic tendency seems to have passed away, and the health of the citizens may be said to be excellent. In another part of this number will be found a report on the disease as it appeared in Louisville.

